# A pilot observational study measuring acute sarcopenia in older colorectal surgery patients

**DOI:** 10.1186/s13104-019-4049-y

**Published:** 2019-01-14

**Authors:** Carly Welch, Carolyn A. Greig, Zaki K. Hassan-Smith, Thomas D. Pinkney, Janet M. Lord, Thomas A. Jackson

**Affiliations:** 1 Institute of Inflammation and Ageing, College of Medical and Dental Sciences, University of Birmingham, Edgbaston, Birmingham, B15 2TT UK; 2 Queen Elizabeth Hospital Birmingham, Mindelsohn Way, Edgbaston, Birmingham, B15 2GW UK; 3 School of Sport, Exercise & Rehabilitation Sciences, University of Birmingham, Edgbaston, Birmingham, B15 2TT, UK; 4 MRC Arthritis Research UK Centre for Musculoskeletal Ageing Research, University of Birmingham, Edgbaston, Birmingham, B15 2TT UK; 5 National Institute for Health Research (NIHR), Birmingham Biomedical Research Centre, University of Birmingham, Edgbaston, Birmingham, B15 2TT UK; 6 Centre for Endocrinology, Diabetes and Metabolism, Birmingham Health Partners, University of Birmingham, Edgbaston, Birmingham, B15 2TT UK; 7 Academic Department of Surgery, Room 29, 4th Floor, Heritage Building, Edgbaston, Birmingham, B15 2TH UK

**Keywords:** Acute sarcopenia, Inflammation, Biomarkers, Aged

## Abstract

**Objective:**

To explore variability in acute changes in muscle mass and function in older patients undergoing elective colorectal surgery, as well as feasibility of measures, in order to refine study processes to inform the protocol for a larger study.

**Results:**

Results are presented for seven participants recruited to this pilot study. It is possible to perform serial measurements of bilateral anterior thigh thickness (BATT) and handgrip strength prior to, within 24 h of surgery, and 1 week postoperatively. Gait speed can be reliably measured preoperatively and at 1 week postoperatively. In this pilot study, BATT and gait speed declined at 1 week postoperatively (median BATT 4.17 cm, 3.47 cm, p = 0.028; median gait speed 0.89 m/s, 0.83 m/s, p = 0.043). Baseline hsCRP correlated with change in BATT (τb = 0.73, p = 0.04) and baseline DHEA-S correlated with change in gait speed (τb = 0.87, p = 0.02). This pilot study has assisted to refine the protocol for our larger study, which will further characterise these changes.

**Electronic supplementary material:**

The online version of this article (10.1186/s13104-019-4049-y) contains supplementary material, which is available to authorized users.

## Introduction

Sarcopenia is defined as reduced muscle mass and function [1]. European Working Group on Sarcopenia in Older People 2 (EWGSOP2) distinguishes acute from chronic sarcopenia [2]. Acute sarcopenia develops within 6 months, normally triggered by a stressor event [2]. Hospitalisation is associated with bedrest and inflammatory response, which can drive catabolism/reduce anabolism, inducing reductions in muscle mass and function; this is exacerbated by age [3, 4]. Precise mechanisms remain unclear. Overview of acute sarcopenia including proposed mechanisms and effectors has been published elsewhere [5].

Colorectal surgery is commonly performed on older adults for localised cancer resection [6, 7] and triggers acute inflammatory response [8]. Patients do not normally experience preoperative functional decline or cachexia [9]. Characterising acute sarcopenia in these patients will help to increase understanding, identify mechanistic pathways, and target interventions, with potential to improve postoperative recovery and longer-term physical function. This study aimed to refine the protocol for a larger study by exploring feasibility and variability of measures.

## Main text

### Methods

#### Study design

Participants were recruited from Queen Elizabeth Hospital Birmingham preoperative assessment clinic during August–October 2017. We included people aged 65 and older, planned to undergo major colorectal surgery (any indication), and able to give informed consent. Exclusion criteria were life expectancy 30 days or less and inability to understand verbal English. Known muscular disorders were not an exclusion criterion. Measurements were taken prior to admission (Visit 1), within 24 h of surgery (Visit 2), and 1 week postoperatively or day of discharge (Visit 3). A single geriatrician with training in procedures performed all assessments (CW). All assessments not possible to perform and reasons for this were recorded.

#### Muscle mass measurement

Quadriceps muscle thickness was measured using B-mode ultrasonography (Venue 50, GE Healthcare, UK). Participants were positioned semi-upright, with a firm wedge below the knees, and advised to relax their muscles. Measurements were taken in transverse plane at mid-point between greater trochanter and knee joint line. Thickness of subcutaneous tissue (SC), rectus femoris (RF), and vastus intermedius (VI), excluding fascia, were measured. Three measurements were taken on each side at each visit and means calculated; a fourth was taken if measurements differed more than 10%. Total bilateral anterior thigh thickness (BATT) (right RF + right VI + left RF + left VI), and BATT:SC ratio (BATT-SCR) were calculated. This method has excellent intra-rater and inter-rater reliability and mean BATT correlates with bioelectrical impedance analysis (BIA) in healthy young adults [10].

#### Muscle strength measurement

Handgrip strength was measured at each visit using a Jamar hydraulic handheld dynamometer (Patterson Medical, Warrenville, IL, USA). Participants were positioned with elbows flexed at 90°, forearms in neutral position and advised to “squeeze as hard as [they] can”. Highest recording of two measurements on each side was used for analysis [11].

#### Physical performance testing

Short Physical Performance Battery (SPPB) was scored at Visit 1; incorporates gait speed, side-by-side/tandem stand, and five chair stands [12]. We initially planned to measure SPPB at all visits; upon consideration of effect of intra-abdominal strain limiting chair stands, gait speed alone was measured at Visit 3. Gait speed is a widely accepted measure of physical performance [1, 13]. A 4 m course was used.

#### Comprehensive geriatric assessment

**Fig. 1:**
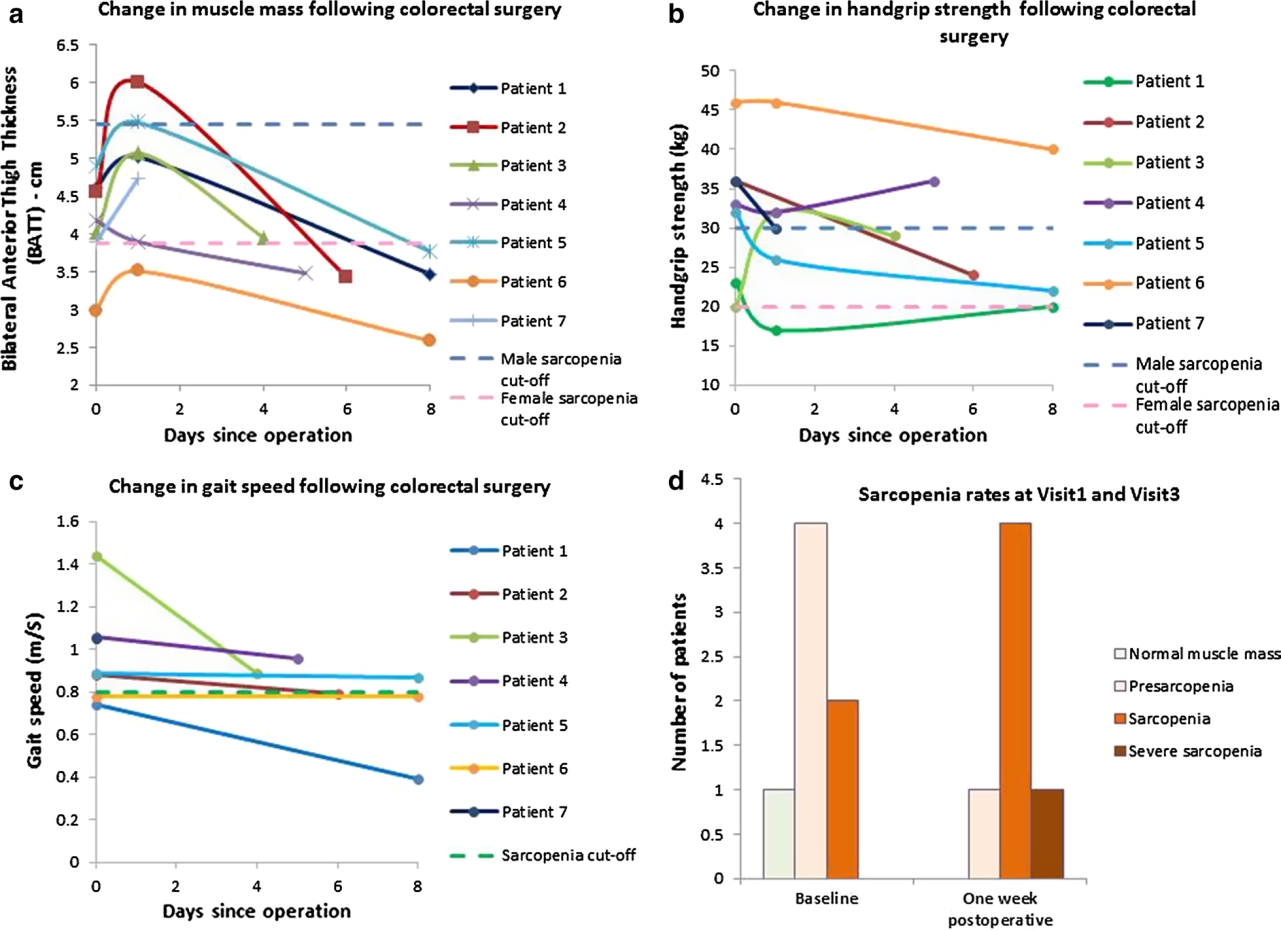
Change in muscle mass and function measured during this pilot study. BATT increased within 24 h of surgery and declined at visit 3 (**a**). Handgrip strength varied widely; two participants experienced increases in handgrip strength and the rest experienced decreases (**b**). Gait speed declined from visit 1 to visit 3 (**c**). Although not statistically significant in this pilot, there was a higher prevalence of sarcopenia at visit 3 compared to visit 1 (**d**). Cut-off points are indicated [BATT 3.86 cm (women), 5.44 cm (men); muscle strength 20 kg (women), 30 kg (men); gait speed 0.8 m/s (both genders)]

During Visit 1, comprehensive geriatric assessment (CGA) was performed by a single geriatrician (CW). This included Mini-Nutritional Assessment Short Form (MNA-SF) [14], Addenbrooke’s Cognitive Examination-III (ACE-III) [15], evaluation of basic [16, 17] and instrumental activities of daily living (ADLs) [18], Geriatric Depression Scale Five-Point (GDS-5) [19, 20], and questioning on falls and admissions within the last year. Comorbidities and symptomatic burden were used to calculate the Geriatric Index of Comorbidity (GI-C) [21]. Frailty was defined by Fried frailty phenotype [22, 23] and Clinical Frailty Scale [24]. Delirium screening with 4AT was performed at each visit [25]. Variables measured were considered potential effectors of acute sarcopenia [5]. These were included to assess feasibility and acceptability of measurement, and document individual differences (Fig. 1).

#### Sarcopenia definition

This study was completed prior to EWGSOP2 dissemination; sarcopenia was defined using initial EWGSOP definition by low BATT (muscle mass) and either low handgrip strength or gait speed [1]. Cut-off values were taken two SDs below young healthy reference population means. Cut-off values were 3.86 cm (women) and 5.44 cm (men) for BATT [10], 20 kg (women) and 30 kg (men) for handgrip strength [13], and 0.8 m/s (both genders) for gait speed [13]. Low BATT alone was defined as pre sarcopenia; severe sarcopenia was defined as all three criteria below cut-offs [1].

#### Enzyme-linked immunosorbent assays (ELISAs)

Blood samples were taken at Visit 1 with serum prepared within 30–60 min of collection and stored at − 80 °C. Cortisol, dehydroepiandrosterone sulfate (DHEA-S), and high sensitivity C-Reactive Protein (hsCRP) (IBL International; Hamburg, Germany), and vitamin D (Immundiagnostik AG 25(OH)-vitamin D direct day ELISA; Bensheim, Germany) were measured by ELISA using commercial kits. Optical density was measured immediately using a photometer at 450 nm (Sci-Tek Instuments Ltd Synergy HT).

#### Data management and analysis

Data were entered into Microsoft Excel 2010, converted to IBM SPSS Statistics 22, and visually assessed for normality. Significance of differences between serial BATT, BATT-SCR, echogenicity, and handgrip strength were calculated using Friedman and post hoc Wilcoxon signed rank tests. Significance of difference in gait speed was calculated using Wilcoxon signed rank testing. Correlation between baseline immune-endocrine markers and changes in BATT, handgrip strength, and gait speed were assessed using Kendall’s correlation. Difference in pre sarcopenia, sarcopenia, and severe sarcopenia rates between Visit 1 and Visit 3 were analysed using Chi squared test. Measurements not obtained at any visit were analysed as missing values.

#### Ethical considerations

Ethical approval was obtained from Wales REC 6 (17/WA/0117). Written informed consent was obtained from participants. Specific optional consent was obtained to consent to remain included in the event they lost capacity to consent during the study. If this were to occur, separate consultee declaration was planned to be obtained from nominated consultees.

### Results

17 patients were approached to participate in this study; eight were recruited. One participant withdrew following Visit 1; data were not analysed. One participant declined handgrip strength testing at Visit 2, and one participant declined Visit 3 assessment. Visit 3 was conducted on median day 7 (IQR 5–8). Additional file 1 shows recruitment and follow-up flowchart for this study. Table 1 displays summary statistics. Mean age was 74.7 years (SD 4.1). 85.7% participants were male. All were White British ethnicity. All participants included in analysis underwent operations for cancer or suspected cancer (e.g. suspicious polyp) (Table 1).

**Table 1:** Demographics and basic participant information

	Mean	SD
Age (years)	74.7	4.1
BMI (kg/m^2^)	24.9	1.8

	Median	IQR
Medications (N)	6	1–6
Falls over last year (N)	0	0–1
Admissions over last year (N)	0	0
Length of stay (days)	7	5–10

	N	Percent of study sample
Male gender	6	85.7
Ethnicity (White British)	7	100
GI-C		
1	1	14.3
2	4	57.1
3	2	28.6
4	0	0
GDS ≥ 2	1	14.3
MNA-SF		
Malnourished	0	0
At risk	2	28.6
Not malnourished	5	71.4
CFS (baseline)		
1–3	6	85.7
4–5	1	14.3
6–8	0	0
Frailty–Fried (baseline)		
Not frail	6	85.7
Pre-frail	1	14.3
Frail	0	0
Surgical technique (open)	6	85.7
Operation		
Ileocecal resection	1	14.3
Right hemicolectomy	4	57.1
Anterior resection	1	14.3
Abdominoperineal resection	1	14.3

The mean age of participants was 74.7 years, with 85.7% being of a male gender. 100% of participants were White British. 85.7% of participants were not frail at baseline by CFS and Fried criteria. Operations performed included ileocecal resection, right hemicolectomy, anterior resection, and abdominoperineal resection. 85.7% were performed as open procedures

#### Changes in muscle mass and function

BATT and BATT-SCR differed between visits (p = 0.006; p = 0.006). Median BATT was 4.17 cm (IQR 3.88–4.39) at Visit 1, 5.01 cm (IQR 3.89–5.47) at Visit 2 (p = 0.028 Visit 1), and 3.47 cm (IQR 3.21–3.81) at Visit 3 (p = 0.028 Visit 1; p = 0.028 Visit 2). Median BATT-SCR was 8.78 (IQR 6.67–13.08) at Visit 1, 5.03 (IQR 4.72–5.32) at Visit 2 (p = 0.018 Visit 1), and 3.94 (IQR 2.82–5.01) at Visit 3 (p = 0.028 Visit 1; p = 0.046 Visit 2). There was no significant change in handgrip strength (p = 0.692). Gait speed declined from median 0.89 m/s (IQR 0.78–1.06) at Visit 1 to median 0.83 m/s (IQR 0.68–0.91) at Visit 3 (p = 0.043). Results are shown in Fig. 1a–c.

#### Acute sarcopenia

Prevalence of sarcopenia at baseline was 28.6%. As assessments were not performed on one participant at Visit 3, incidence of acute sarcopenia at Visit 3 was 75%. Overall prevalence of sarcopenia at Visit 3 was 83.3%. One participant, who had pre sarcopenia at Visit 1, developed severe sarcopenia. Changes were not statistically significant (p = 0.558). Results are shown in Fig. 1d.

#### Immune-endocrine markers

**Fig. 2:**
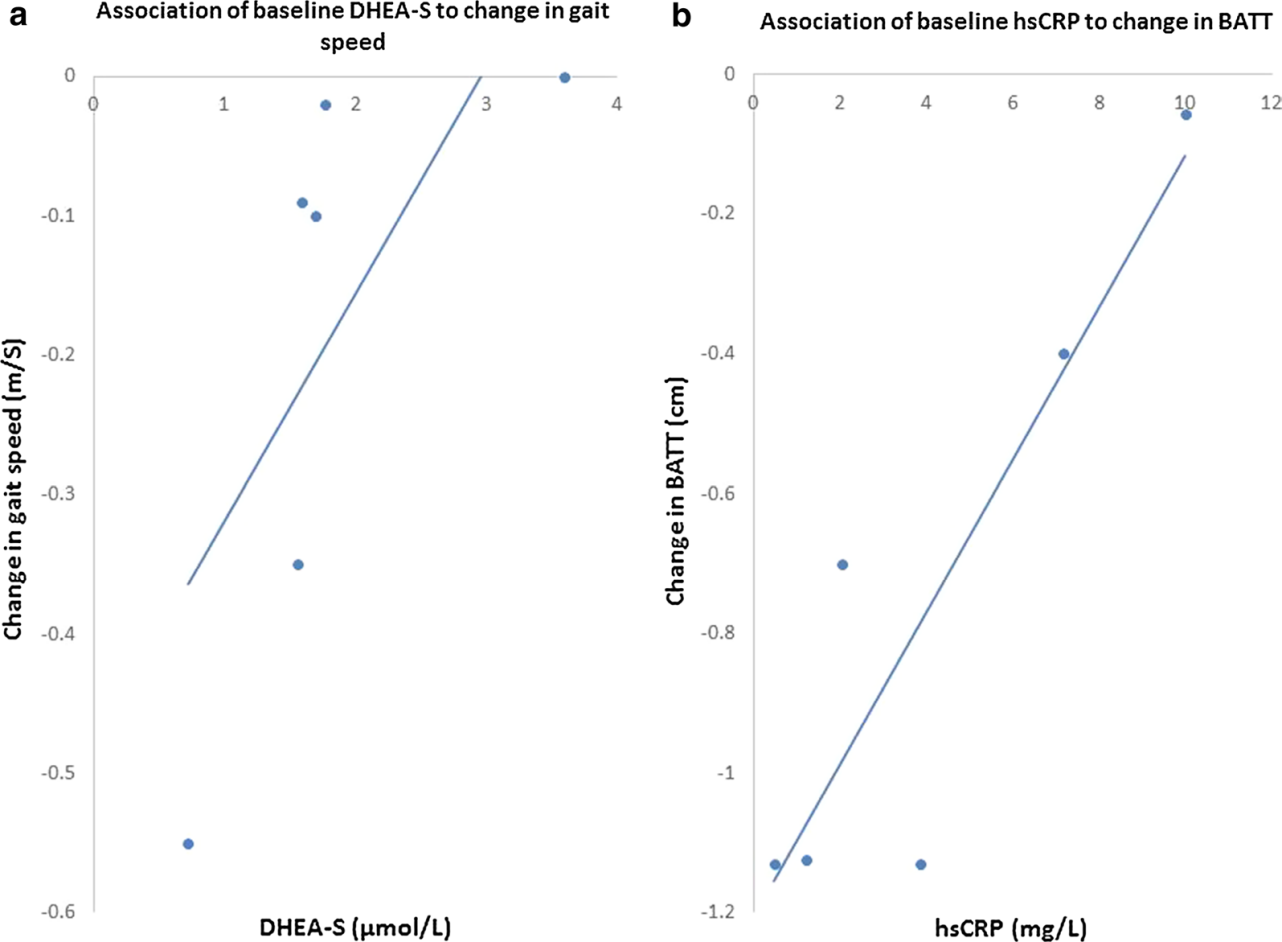
Association of DHEA-S to change in gait speed and hsCRP to change in BATT. There was a positive association between baseline DHEA-S and change in gait speed (τb = 0.87, p = 0.02) (**a**) and a positive association between baseline hsCRP and change in BATT (τb = 0.73, p = 0.04) (**b**)

There was a positive association between baseline DHEA-S and gait speed change (τb = 0.87, p = 0.02) (Fig. 2a), and between baseline hsCRP and BATT change τb = 0.73, p = 0.04) (Fig. 2b). No other significant associations were identified.

#### Feasibility and acceptability of measurements

It was possible to perform all muscle assessments in all participants at Visit 1. BATT was measured at each visit with participants, although one participant declined all assessments at Visit 3. One participant declined handgrip strength assessment at Visit 2. SPPB was measured at baseline; only gait speed was measured at Visit 3, due to potential harm from abdominal strain in performing chair stands. Nutritional, mood, functional, and comorbidity assessment were performed in all participants at Visit 1. Cognitive screening was initially performed, but following withdrawal of one participant due to burden of assessment, a decision was made to remove formal cognitive testing. Participants expressed they found all other assessments acceptable. No participant exhibited loss of capacity, but all participants consented to ongoing inclusion in the event they should have lost capacity during the study.

### Discussion

Prevalence of sarcopenia at Visit 1 was similar to previous studies in hospitalised older patients [26–29]. Reduction in BATT and gait speed, and increased prevalence of sarcopenia at Visit 3 compared to Visit 1 was demonstrated. Muscle strength change showed greater variability between participants. The next stage of our research will characterise a larger cohort, including elective and emergency admissions, and include three-month follow-up. We have shown that measurement of these variables is feasible in an acute care setting. Due to perceived burden of ACE-III, and following further public involvement, this will not be measured in our main study. This should enable time to focus on nutritional and physical function assessment, and comparison measurements of muscle mass with BIA.

BATT increased at Visit 2 in all but one participant; BATT-SCR reduced across visits for all participants. This suggests increased BATT was associated with increased SC thickness. We consider increased SC thickness more likely represents oedema than increase in fat tissue. Similar findings have been demonstrated in critical care patients following cardiothoracic surgery, with quadriceps muscle thickness correlating with fluid balance [30]. However, changes in fluid balance alone may be insufficient to explain this. Fluid shifts may have been exacerbated by systemic inflammation and increased vascular permeability postoperatively [31]. Considering increases in BATT within 24 h of surgery may represent heightened systemic inflammation, this should not be disregarded as a benign process. If acute increase in BATT relates to increased inflammation, this could herald increased likelihood of developing acute sarcopenia. Our planned larger study will assess if rate of change to Visit 2 predicts rate of change to Visit 3.

Baseline hsCRP correlated with change in BATT to Visit 3, suggesting older adults with lower baseline inflammation were at greater risk of acute reductions in muscle size. This is converse to associations demonstrated in chronic sarcopenia; higher levels of hsCRP and proinflammatory cytokines have been demonstrated in older adults with sarcopenia [32] although, a positive association between hsCRP and lean appendicular mass was demonstrated in a large cohort study [33]. Baseline DHEA-S levels correlated with change in gait speed; age-related reduced DHEA-S has been shown to correlate with reduced physical performance [34]. We will further evaluate this in our larger study; a proven association may suggest exogenous DHEA-S could be used to protect older adults from acute declines in physical performance.

Our main study will fully characterise acute muscle changes, and acute sarcopenia in elective and emergency patients. This will include CGA, particularly nutritional and physical function assessment, and measurement of biomarkers of immune-endocrine function, as guided by our pilot results. Follow-up will be conducted to assess if acute changes in muscle mass and function are predictive of change in physical function at 3 months.

## Limitations

Firstly, we recognise that, although tests of statistical significance were performed, this pilot study was not powered to identify changes in muscle mass or function. We fully acknowledge that no definite conclusions can be made from this study alone. However, we consider these results may be of value in future research and, potentially, used in a systematic review. Our results have reaffirmed the need for our larger study, and assisted with power calculations.

An important characteristic that differed from expected population within this study was gender. Percentage of elective colorectal surgery procedures performed on females has been reported as 41.7–56.2% [35–37]. This is important as muscle echotexture, muscle: SC tissue ratio, and muscle force generation and relaxation are reported to differ between genders [38]. We acknowledge that analysis of genders together may have confounded results.

All assessments were performed by a single investigator. Although this ensured standardisation of technique, this may have induced systematic error. Handgrip strength is recommended to be measured with the participant sat upright and elbow flexed at 90°. Measurements were taken in this position wherever the participant was able to sit out; however, in some circumstances at Visit 2 this was not possible. In these cases, handgrip strength was assessed with the participant in a semi-upright position in bed with elbow flexed as close to 90° as possible. This may have affected results at this time point.

Lastly, there is some evidence that position of the participant when measuring quadriceps thickness can affect results [39]. Measurement has been recommended with the participant lying supine, as most objectively standardised position [40]. However, some participants were unable to lie flat at Visit 2, due to increased intraabdominal strain and postoperative nausea. Therefore, ultrasound assessments were performed in a semi-upright position. An attempt was made to standardise this position as much as possible, but this was not assessed objectively; there may have been some variability in bed position between visits. Measurements taken in the reference population were taken in a similar position [10].

## Additional file

**Additional file 1.** Recruitment and follow-up flowchart. Flowchart to clearly demonstrate recruitment, enrolment, and follow-up of participants within this research study.

## Abbreviations

4AT: 4 “A”s test
ADL: activity of daily living
BATT: bilateral anterior thigh thickness
BATT-SCR: bilateral anterior thigh thickness: Subcutaneous tissue ratio
BIA: bioelectrical impedance analysis
CFS: Clinical Frailty Scale
DHEA-S: dehydroepiandrosterone sulfate
ELISA: enzyme-linked immunosorbant assay
GDS-5: Geriatric Depression Scale 5
GI-C: Geriatric Index of Comorbidity
hsCRP: high sensitivity C-Reactive Protein
IQR: interquartile range
MNA-SF: Mini Nutritional Assessment (Short Form)
RF: rectus femoris
SC: subcutaneous tissue
SPPB: Short Physical Performance Battery
VI: vastus intermedius
