# Supplementary material for: A pilot observational study measuring acute sarcopenia in older colorectal surgery patients
Source: BMC Res Notes. 2019 Jan 14;12:24. doi: 10.1186/s13104-019-4049-y (PMC6332645; doi:10.1186/s13104-019-4049-y)
Supplement: Supplementary file 1 — Additional file 1. Recruitment and follow-up flowchart. Flowchart to clearly demonstrate recruitment, enrolment, and follow-up of participants within this research study. [file 13104_2019_4049_MOESM1_ESM.docx]

**Recruitment and follow-up flowchart**

Approached to participate (n=17)

Excluded (n=9)

♦ Declined to participate (n=9)

## Enrollment =8

Excluded (n=1)

♦ Voluntary withdrawal from study (n=1)

## Follow-Up (visit 2) = 7

## Follow-Up (visit 3) = 6

## Analysis = 7

Analysed (n=7)
♦ Excluded from analysis (n=0)

♦ Declined follow-up assessments (n=1)

(Included in analysis at visits 1 and 2)
